# The Expression of Insulin in the Central Nervous System: What Have We Learned So Far?

**DOI:** 10.3390/ijms24076586

**Published:** 2023-04-01

**Authors:** Tamara Dakic, Tanja Jevdjovic, Iva Lakic, Aleksandra Ruzicic, Nebojsa Jasnic, Sinisa Djurasevic, Jelena Djordjevic, Predrag Vujovic

**Affiliations:** Department for Comparative Physiology and Ecophysiology, Institute for Physiology and Biochemistry Ivan Djaja, Faculty of Biology, University of Belgrade, Studentski Trg 16, 11000 Belgrade, Serbia

**Keywords:** brain-derived insulin, hypothalamus, hippocampus, cerebellum, cerebral cortex, olfactory bulb, growth and development, glucose homeostasis, Alzheimer’s disease

## Abstract

After being discovered over a century ago, insulin was long considered to be a hormone exclusively produced by the pancreas. Insulin presence was later discovered in the brain, which was originally accounted for by its transport across the blood-brain barrier. Considering that both insulin mRNA and insulin were detected in the central nervous system (CNS), it is now known that this hormone is also synthesized in several brain regions, including the hypothalamus, hippocampus, cerebral and cerebellar cortex, and olfactory bulb. Although many roles of insulin in the CNS have been described, it was initially unknown which of them could be attributed to brain-derived and which to pancreatic insulin or whether their actions in the brain overlap. However, more and more studies have been emerging lately, focusing solely on the roles of brain-derived insulin. The aim of this review was to present the latest findings on the roles of brain-derived insulin, including neuroprotection, control of growth hormone secretion, and regulation of appetite and neuronal glucose uptake. Lastly, the impairment of signaling initiated by brain-derived insulin was addressed in regard to memory decline in humans.

## 1. Introduction

A large number of review articles have already addressed the actions of insulin within the central nervous system (CNS). However, to the best of our knowledge, none of them has focused exclusively on the functions of insulin produced in the CNS. Therefore, this review article consists of two major parts; the first offers a short overview of some of the studies that documented insulin expression in various brain regions, and the second represents the collection of recently published data describing the mechanisms controlling insulin expression in the brain and its roles. The fact that neuronal insulin expression is observed not only in rodents and humans but in invertebrates as well [1] implies that the roles of brain-derived insulin are evolutionarily conserved and, as such, are worthy of thorough examination.

## 2. Overview of Studies Demonstrating Insulin Expression in the Central Nervous System (CNS)

After being discovered over a century ago, insulin was long considered to be an exclusively β-pancreatic cell-derived hormone that lowered glucose levels upon its secretion in the systemic circulation [2]. The research that followed its discovery was therefore focused on describing insulin peripheral effects and their applications in the treatment of diabetes. It has long been known that insulin stimulates glucose uptake by skeletal muscles [3] and white adipose tissue [4]. Additionally, insulin regulates liver glucose and lipid metabolism [5].

The presence of insulin in the brain of adult rats was initially observed more than half a century after its initial discovery. Namely, it was reported that insulin “indistinguishable” from that typically found in the pancreas was found in extracts of the whole rat brain [6]. Moreover, obesity was also proven to affect brain insulin content. Namely, the concentrations of insulin extracted from the olfactory bulb, hypothalamus, hippocampus, cerebral cortex, amygdala, midbrain, and hindbrain were significantly lower in obese (fa/fa) than in lean (Fa/Fa) Zucker rats [7].

**Figure 1:**
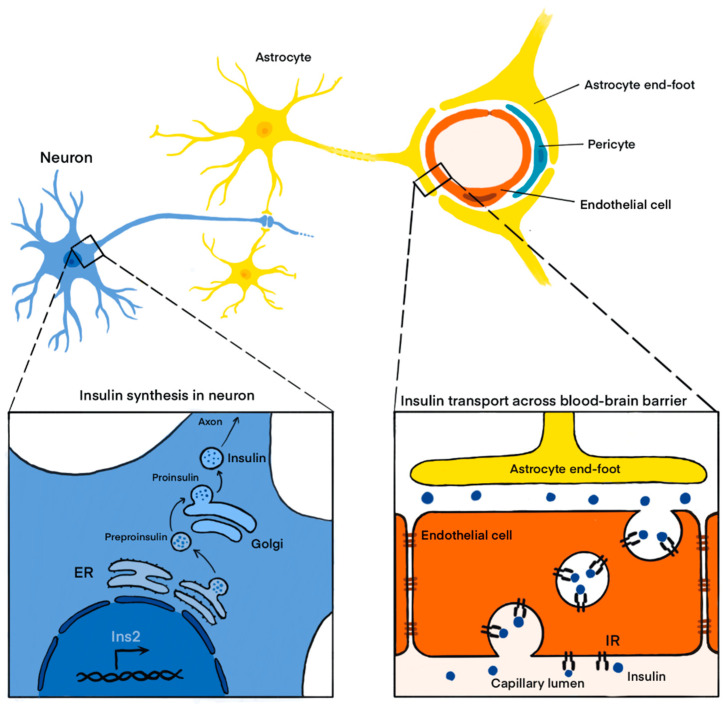
Insulin expression and presence in the central nervous system. Insulin synthesized in the pancreas is secreted into the systemic circulation and transported to the brain across the blood-brain barrier by receptor-mediated transcytosis. In addition, insulin is synthesized in neurons. *Ins2*—insulin 2; IR—insulin receptor; ER—endoplasmic reticulum.

However, the insulin immunoreactivity in the CNS was initially accounted for by its transport across the blood-brain barrier (BBB). The transport of insulin from the blood into the cerebrospinal fluid (CSF) was documented even before its presence in the brain parenchyma. The study conducted on dogs showed that intravenous insulin administration resulted in increased CSF levels of this hormone [8]. The transport of insulin across the BBB was later characterized as saturable receptor-mediated transcytosis [9,10]. A debate on the origin of insulin detected in the CNS started not long after its presence was detected in several brain regions. Still, it was not until more sophisticated molecular biology techniques had been developed that it became evident insulin not only crossed the BBB but was also expressed within the CNS (Figure 1).

### 2.1. In Vitro Studies Confirming the Expression of Insulin in Neurons

A body of evidence suggesting that in addition to the pancreas, insulin is also expressed in the brain has been growing for almost four decades. For example, insulin immunoreactivity was detected in cells cultured from rats [11,12] and mouse fetal brains [13] in the early 1980s. Additionally, the presence of insulin immunopositivity was shown in rabbit primary neuronal cell cultures [14]. Subsequent studies confirmed that, in addition to the protein itself, insulin was present in the cultured neurons at the mRNA level as well [14,15,16,17]. It is important to note that insulin was detected in cultured neurons but not in glial cells [16]. Some of the earlier studies also demonstrated insulin secretion from primary rats [18] and rabbits [17] cultured neuronal cells after inducing membrane depolarization by decreased efflux of potassium ions in the presence of calcium [18].

More recent in vitro studies not only reconfirmed early findings that insulin was expressed in neurons but also offered more information on the mechanisms regulating this process. Molnár et al. [19] showed that *Ins2* mRNA expression took place in the perisomatic cytoplasm of inhibitory (GABAergic) interneurons of rat cerebral cortex and found that lowering glucose concentrations in the medium to the levels normally found in the brain extracellular fluid during hypoglycemia decreased the number of *Ins2* mRNA molecules detected per neurogliaform cell (NGFC). On the other hand, the addition of glucose or glibenclamide (hypoglycemic drug) to the medium increased insulin concentration in the neocortical slices. Furthermore, the insulin secretion from NGFC was found to be dependent on dendritic Ca^2+^ entry.

A different study showed that a glucose-induced increase in insulin expression in NGFC occurred via the activation of glucagon-like peptide-1 receptor (GLP-1R) [20]. It was demonstrated not only that *Ins2* mRNA and *Glp1r* mRNA were co-expressed in these GABAergic neocortical interneurons but that the expression of both of these genes was upregulated by high extracellular glucose concentration. Functional expression of GLP-1R in NGFC was confirmed by showing that GLP-1 treatment had a reversible effect on their electrophysiological properties which was prevented by the application of exendin-4, a GLP-1R antagonist. Collectively, these findings indicate that similar to pancreatic β cells, insulin expression in neocortical inhibitory interneurons is stimulated by endogenous incretins such as GLP-1.

In addition to neocortical, glucose was shown to stimulate insulin expression in the mouse hypothalamic neuronal cell lines (mHypoE-39 and mHypoE-46) via a similar mechanism [21]. Namely, the levels of *Ins2* mRNA were significantly upregulated after the treatment of hypothalamic neurons with a hyperglycemic solution. A similar response was observed after the treatment of these cells with forskolin (an activator of adenylate cyclase) and exendin-4. However, the responses induced by forskolin and exendin-4 were biphasic, with *Ins2* mRNA expression being initially increased above but ultimately reduced below the control level. These findings showed that changes in glucose and cyclic adenosine monophosphate (cAMP) levels affected insulin expression in a certain subset of hypothalamic neurons. Considering that these neurons, in addition to insulin and glucose transporters, also express glucokinase, one might hypothesize that changes in hypothalamic insulin secretion may serve as an output signal to downstream neurons involved in the regulation of energy homeostasis. However, additional analyses are needed in order to confirm this hypothesis.

Another study on a hypothalamic neuronal cell line confirmed that insulin was produced in mHypoE-39 cells but also provided additional insights into which other molecules took part in regulating insulin expression in the CNS [22]. This study uncovered that the treatment with Wnt3a, a signaling molecule known for regulating pleiotropic cellular functions [23], significantly increased *Ins2* gene expression and insulin secretion. Moreover, neurogenic differentiation 1 (NeuroD1), one of the transcription factors that regulate insulin gene expression, was also induced by Wnt3a treatment in a time- and dose-dependent manner. Additionally, it was shown that treatment by a glycogen synthase kinase-3 (GSK3) inhibitor also increased the expression of both insulin and NeuroD1 in mHypoE-39 hypothalamic cells. These findings initially observed in cultured hypothalamic cells were subsequently validated in vivo. Namely, the knockdown of NeuroD1 not only prompted a decrease in basal *Ins2* expression but also suppressed Wnt3a-induced *Ins2* expression. Furthermore, the Wnt3a hypothalamic injections increased the expression of *NeuroD1* and *Ins2* in mice in a manner similar to that observed in vitro.

### 2.2. In Vivo Studies Confirming the Expression of Insulin in the CNS

Despite their multitude, the studies conducted on cultured mammalian cells were not sufficient to prove that insulin expression unequivocally takes place in vivo in the brain. The first study reporting insulin presence in the rat brain was conducted by Havrankova et al. in 1978 [6]. Later, insulin immunoreactivity was confirmed in different brain regions in rats [24,25,26], mice [24], rabbits [17], and humans [27,28]. However, insulin’s presence in the brain remained controversial for a long time. One of the first steps toward resolving this controversy was the observation that, in neonatal rabbits, there was a positive correlation between brain and CSF insulin concentrations in the absence of a relationship between serum and CSF concentrations [29]. This indirectly implied that in addition to the transport across the BBB, the neonatal brain independently contributed to the amount of insulin found in the CSF. Additionally, studies that showed the presence of insulin mRNA corroborated its synthesis in fetal, neonatal, and adult brain tissue [30,31,32]. Studies conducted on rodents showed that, unlike the duplicated *Ins1* gene, the ancestral *Ins2* gene was expressed in fetal, neonatal, and adult rat brains [30,32]. It should be mentioned that there are two insulin genes in rodents. *Ins1* was retroposed from the partially processed *Ins2* mRNA, while the *Ins2* gene is the ortholog of the human *INS* gene [33].

A study conducted on mice showed that insulin was broadly expressed throughout developing and adult mice brains [32]. The presence of insulin was documented at the levels of *Ins2* mRNA, mature insulin, and C-peptide in the cerebellum, cerebral cortex, anterior olfactory bulb, cerebellar Purkinje neurons, and most prominently in the hippocampus. Quantitative analysis showed that the rate of hippocampal insulin expression was lower than that in the pancreas. This finding, in addition to the observation of close contacts between neurites and perikaryia or neurites of neurons positively stained for insulin [11] and fact that *Ins*^−/−^ mutants do not show significant pathological changes under physiological conditions [34] indicates that brain-derived insulin most likely acts either as an autocrine and/or paracrine signaling molecule, unlike the pancreatic which is secreted into the systemic circulation and thus takes part in the long-distance communication in a body. Mehran et al. [32] also discovered regions of active transcriptions around the *INS* gene in several regions of the human brain, including the hippocampus, cerebral cortex, and cerebellum. Table 1. summarizes representative publications confirming the neuronal insulin expression in vitro and in vivo.

## 3. Actions of Insulin Expressed in the CNS

The expression of insulin receptors (IR) in the CNS was first documented almost half a century ago [53]. It is now known that both short (IR-A) and long (IR-B) isoform of this receptor is expressed in the hypothalamus, hippocampus, cerebral cortex, and cerebellum [54,55], the brain regions also associated with the production of insulin. In regard to the IR expression at the cellular level, it was shown that, unlike neurons that only express IR-A, astrocytes express both IR-A and IR-B [56,57]. Ever since the transport of insulin across the BBB and the expression of functional IR in the CNS was documented, attempts have been made to learn more about the effects of insulin on the brain. In line with that, numerous actions of insulin in the CNS have been described so far. It has been known that insulin takes part in controlling food intake and body weight [58]. In addition to that, insulin actions are also essential for proper neuronal development and survival [59], cognition [60], brain cholesterol synthesis [61], hepatic glucose production [62], lipolysis and lipogenesis [63], and even reproductive competence [64]. Moreover, it was also shown that impairment in insulin signaling could trigger depression- and anxiety-like behaviors [65]. Although new roles of insulin keep emerging, it is still mainly unknown which of them can be attributed to brain-derived and which to pancreatic insulin, or whether their actions in the CNS overlap and to what extent.

In the remainder of this manuscript, we aimed to overview the data yielded mainly by more recent studies concerning specifically the actions of insulin synthesized in various brain regions and how their impairment may be associated with some neurodegenerative diseases.

### 3.1. Brain-Derived Insulin Has Neuroprotective Effects

Some of the earliest studies concerning the role of insulin produced in neurons were performed on cultured cells. One of the first roles of neural insulin to be observed was that it promoted neurofilament distribution and axonal growth via mitogen-activated protein kinase (MAPK) phosphorylation in cultured rat fetal neurons [35,36]. Cultured neurons from fetal rat brains incubated in an insulin-free medium were first shown to contain *Ins2* mRNA and preproinsulin, a biosynthetic precursor of insulin. Subsequently, neurofilament immunoreaction was detected in the somata, dendrites, and axons of these neurons. Lastly, the treatment with either insulin antibody or the inhibitor of insulin receptor tyrosine kinase activity was shown to result in neurite retraction rendering neurons hypertrophic and vacuolated. Inhibition of MAPK, but not phosphatidylinositol 3-kinase (PI3K), also resulted in shortening and/or complete retraction of neuritis and rounding of neurons, which suggested that insulin effects on neurofilament distribution to the axons were accomplished via MAPK activation.

A study with a similar design also showed that insulin of neural origin promoted neural differentiation and growth [66]. Moreover, it was demonstrated that in vivo interference with embryonic insulin signaling by blocking insulin receptors increased apoptosis during early neurulation and thus showed that proinsulin produced by neuroepithelial cells promoted cell survival during this stage of embryonic development [67]. In another study, proinsulin was shown to decrease the expression of neuroinflammation markers through the activation of protein kinase B (Akt) pathways and lower levels of tumor necrosis factor-α (TNF-α) and interleukin 1β in the mouse hippocampus [68]. Of note, these effects detected at the molecular level correlated with improved cognitive performance. Collectively, these findings indicate that centrally produced (pro)insulin exhibits neuroprotective and anti-inflammatory properties.

### 3.2. Fasting-Induced Increase in Hypothalamic Insulin Expression Appears Not to Be Associated with Facilitating Neuronal Glucose Uptake

The distinctiveness of mechanisms controlling peripheral and central insulin expression became evident in one of our studies which examined the effects of short-term fasting on insulin expression in the rat hypothalamus. Our study showed that six-hour fasting increased both *Ins2* mRNA expression and insulin content in the hypothalamic parenchyma, while the concentrations of circulating glucose and insulin were expectedly to decrease [50]. Furthermore, immunopositivity for preproinsulin was observed in the neurons of the hypothalamic periventricular nucleus (PEV) and the ependymal cells lining the roof of the third cerebral ventricle. These two cell types were already recognized as the sources of insulin within the CNS. The presence of *Ins2* mRNA was first detected in the neurons of PEV almost four decades ago [40], while the epithelial cells, such as those of choroid plexus (EChP), were identified as the source of insulin more recently [38]. Namely, Mazucanti et al. [38] found that *Ins2* mRNA, mature insulin, and C-peptide were all present in EChP. Furthermore, they demonstrated that insulin secretion from primary cultured mouse EChP was stimulated by serotonin (5-HT). More precisely, it was shown that activation of the 5HT_2C_ receptor by serotonin treatment resulted in the opening of IP_3_-gated Ca^2+^ channels in the endoplasmic reticulum. This resulted in Ca^2+^ being mobilized from intracellular storage, which consequently led to insulin secretion. The role of serotonin in the secretion of insulin from EChP was indirectly corroborated in vivo by showing that serotonin depletion in the dorsal raphe nucleus downregulated insulin expression in ChP. Our unpublished data showed that, such as insulin, serotonin content in the hypothalamus was also elevated following the six-hour fast. Additionally, hypothalamic insulin increment was observed following microdialysis administration of dexfenfluramine, a potent 5-HT stimulator [69]. This suggests that in addition to basal conditions, serotonin may take part in stimulating the secretion of brain insulin during metabolically challenging states such as fasting.

In regard to the ependymal cells in our study, strong granular proinsulin immunopositivity was observed in the apical portion of cuboidal ciliated cells in the region surrounding the lumen of the third ventricle. The intracellular location of proinsulin-containing granules implies that insulin produced in these cells was secreted into the CSF rather than being taken up from it. This conclusion was supported by the fact that, unlike circulating insulin which was lowered, the CSF insulin levels were at the control level despite the short-term food deprivation [50].

We then sought to decipher the adaptive significance of the short-term fasting-induced increase in hypothalamic insulin expression. Considering that fasting represents metabolic strain for the organism as a whole, we wanted to investigate whether this phenomenon was related to maintaining glucose homeostasis within the hypothalamus. It was found that short-term fasting markedly increased the amount of endothelial 55 kDa isoform of glucose transporter 1 (GLUT1) and neuronal GLUT3. The levels of GLUT2, whose presence was detected in neurons, ependymocytes, and tanycytes, were also elevated [70]. However, the absence of co-expression of these membrane transporters with the activated insulin receptor suggested that the actions of this locally produced insulin were unlikely associated with the regulation of glucose-facilitated diffusion in the hypothalamus during fasting.

A further attempt to determine the role of fasting-promoted increase in the hypothalamic insulin expression was made by looking into how short-term fasting affected signaling pathways typically activated by this hormone [71]. We found that the hypothalamic content of total and activated insulin receptor substrates 1 and 2 (IRS1/2), PI3K, and the mammalian target of rapamycin (mTOR) was unaltered. However, the levels of phosphorylated Akt1/2/3 were decreased, unlike those of activated extracellular signal-regulated kinases (ERK1/2), which were increased. Moreover, activated ERK1/2 was co-expressed with activated insulin receptors in the nucleus arcuatus, which suggested that the ERK activation in the hypothalamus was at least partially initiated by the centrally produced insulin during short-term fasting.

Lastly, we wanted to examine how fasting of the same duration would affect hypothalamic insulin expression in female rats during different phases of the estrus cycle [52]. Following the six-hour food deprivation, *Ins2* mRNA expression and insulin content were not elevated as previously observed in male rats, but they were not reduced either. Both of these parameters remained unaltered in both proestrus and diestrus, despite circulating insulin being significantly lowered. Similar to findings observed in male rats, insulin immunopositivity was detected in the PEV neurons and the ependymal cells at the top of the third cerebral ventricle in females. When taken together, these data indicate that control of insulin expression in the hypothalamus during short-term fasting is a sex-specific process. However, hypothalamic insulin expression in both sexes appeared to be distinctly regulated during fasting than that in the pancreatic β cells.

### 3.3. Stimulation of Insulin-Producing Neurons in Dorsal Vagal Complex Results in Increased Appetite

Another recent study also shed light on the complexity of centrally-produced insulin expression patterns and actions [72]. First of all, this study showed that in addition to hypothalamic nuclei, such as nucleus arcuatus and paraventricular nucleus (PVN), the *Ins2*-promoter-containing cells were also found in the nucleus of the solitary tract and the dorsal vagal complex (DVC). Furthermore, this study showed that insulin expression in the brain was affected by dietary interventions in a region-specific manner. For example, eight-week exposure to a high-fat diet (HFD) decreased *Ins2* mRNA expression in the hypothalamus while simultaneously increasing it in DVC. This finding suggests that the role of insulin produced in the hindbrain becomes more evident under metabolically stressful situations such as prolonged exposure to HFD. In contrast to the anorexic effects that insulin typically exhibits in the hypothalamus, this study revealed that stimulation of DVC insulin-producing neurons results in an acute increase in appetite. Namely, optogenetic stimulation of DVC insulin-producing neurons resulted in increased food intake, which peaked one hour after the onset of stimulation without affecting long-term appetite. Ventricular application of a highly specific insulin receptor antagonist blocked the aforementioned stimulating effects of DVC insulin-producing neurons on feeding.

### 3.4. Insulin Produced in the Paraventricular Nucleus Stimulates Growth Hormone Secretion

A major breakthrough in unveiling the physiological role of hypothalamus-derived insulin came from the study of Lee et al. [51]. Not only did the authors describe the properties of the hypothalamic insulin-producing neurons in mice, but they also showed that insulin synthesized in this region controls the secretion of one of the anterior pituitary hormones. In this study, *Ins2* mRNA, proinsulin, and mature insulin were colocalized within the same subset of neurons in the PVN. Proinsulin was mainly found in the neuronal cell bodies. In addition to that, proinsulin was also detected in the ependymal cells lining the third ventricle and, to a lesser extent, in the ameboid microglia. Unlike proinsulin, C-peptide, which connects the A and B chains of proinsulin and is secreted together with insulin, was located in neurosecretory nerve terminals in the external zone of the median eminence (ME). This suggests that C-peptide and matured insulin are transported from the somata of PVN insulin-producing neurons through axonal projections to their axon terminals in the ME.

Most of the insulin-producing PVN neurons (90%) examined in this study were shown to also synthesize corticotropin-releasing hormone (CRH), while somatostatin was found in only about 20% of insulin-producing neurons. Moreover, CRH was extensively colocalized with C-peptide within the same large dense-core vesicles in the axon terminals in the ME. It is noteworthy that the expression patterns of insulin and CRH genes were reciprocal during the eight-hour exposure to restraint stress. While *Crh* mRNA expression proved to be biphasic, peaking half an hour and eight hours after the onset of restraint, hypothalamic *Ins2* mRNA levels were continuously decreased during the exposure to restraint.

The proximity of PVN insulin-producing neuronal axon terminals to the ME led the authors of this study to examine whether PVN-derived insulin might be involved in the control of anterior pituitary hormone secretion. The only pituitary hormone affected by the knockdown of PVN insulin was growth hormone (GH). Namely, both pituitary *Gh* mRNA expression and the serum GH concentration were lowered after the insulin gene had been knocked down in the PVN. Considering that hypothalamic expression of growth hormone-releasing hormone (GHRH) and somatostatin was unaltered by PVN insulin knockdown, it was concluded that hypothalamic insulin-regulated GH secretion independently of these two hormones. Moreover, PVN insulin knockdown significantly suppressed the pituitary phosphorylation of Akt, the kinase known to be a major target of insulin signaling. Ultimately, the importance of PVN insulin in promoting GH secretion was confirmed in PVN insulin-knockdown young mice. Six weeks after hypothalamic *Ins2* mRNA expression had been downregulated, a significant reduction in body length (without changes in food intake and body weight) of these mice was documented in comparison to the controls of the same age [51]. This finding is particularly relevant given that growth in children can be hampered by exposure to stressful stimuli [73].

### 3.5. In Utero Alcohol Exposure Decreases Insulin Expression in the Cerebellum and Thus Impairs Insulin-Mediated Neuronal Glucose Uptake

The significance of centrally produced insulin for normal brain development was examined in a study conducted by Monte et al. [45]. They studied the effect of in-utero alcohol exposure on insulin expression and signaling in the early postnatal rat cerebellum, one of the brain regions most susceptible to ethanol neurotoxicity. It was found that chronic exposure to ethanol during gestation caused cerebellar hypoplasia with loss of neurons due to increased apoptosis. On the molecular level, exposure to ethanol downregulated the expression of insulin mRNA both in the cerebellum as a whole and in the primary culture of granule neurons isolated from the cerebella of ethanol-exposed pups. In regard to its receptor, there were no significant differences in the levels of insulin receptor mRNA in either ethanol-exposed cerebella in general or neurons isolated from that brain region. However, the levels of the intrinsic insulin receptor tyrosine activity were reduced following the gestational ethanol exposure.

It had already been known from earlier studies that ethanol-induced inhibition of insulin-stimulated tyrosine phosphorylation of insulin receptor and IRS1 and the subsequent impairment of downstream PI3K/Akt signaling results in decreased neuronal survival [74]. Among other targets, Akt was shown to phosphorylate protein tyrosine phosphatase 1b (PTP1b) and thus decrease its ability to dephosphorylate insulin receptors [75]. Therefore, the reduction in the Akt-mediated negative regulation of PTP1b resulted in increased activity of this enzyme despite the fact that the levels of *PTP1b* mRNA levels were not changed within the cerebellum. Considering the aforementioned, decreased neuronal sensitivity to insulin following exposure to ethanol can be accounted for by PTP1b-mediated dephosphorylation of insulin receptor.

The implications of ethanol-induced decrease in insulin expression in the cerebellum were also analyzed within the context of glucose uptake and utilization. Chronic gestational exposure to ethanol reduced the levels of *Glut4* mRNA [45] and the cerebellar amount of this glucose transporter, whose translocation to the cell membrane is known to be insulin-dependent [76]. Consequently, both basal and insulin-induced glucose uptake was decreased in neuronal cultures cultivated from ethanol-exposed cerebella. Reduced glucose uptake was reflected in the ATP levels being consistently lowered in these cells in comparison to the control values. These findings are in line with a previous finding that the expression of the insulin-responsive gene coding for glyceraldehydes-3- phosphate dehydrogenase (GAPDH) was significantly decreased [74]. Taken together, the data from various studies of gestational exposure to ethanol suggest that neuronal glucose uptake and utilization, and consequently neuronal survival in the developing cerebellum are, at least partially, mediated by insulin expressed within this brain region.

### 3.6. The Impairment in Insulin Expression and Signaling in the Brain Is Associated with Memory Decline

The effects of treatments mimicking the stress exposure on insulin and insulin receptor expression in the brain were described in the study of Osmanovic et al. [48]. The authors found that, following the chronic administration of exogenous corticosterone, the expressions of both insulin and insulin receptors were reduced in the cerebral cortex. However, the treatment which mimicked the exposure to chronic stress resulted in elevated tau protein mRNA expression within the same brain region. Of note, these molecular changes positively correlated with a decline in working and reference memory. All of these findings are in line with the fact that Alzheimer’s disease is often associated with insulin-deficient and/or insulin-resistant brain states [77,78]. Collectively, these data point to the contribution of brain-derived insulin in preserving cognition, the process which is also known to be compromised by impaired transport of pancreatic insulin across the BBB [79] and proper functionality of IR in the CNS [80].

As previously mentioned, studies have shown that insulin signaling impairment is associated with pathologies such as Alzheimer’s disease [81]. Moreover, insulin concentration is reduced in the CSF of patients with Alzheimer’s disease [82], while decreased insulin levels were found post-mortem in the brain parenchyma of patients who suffered from sporadic Alzheimer’s disease [44]. One of the first studies that provided insight into how hippocampal insulin expression and secretion was affected by amyloid-β_1–42_ (Aβ_1–42_) was that of Nemoto et al. [49]. They first reconfirmed that both *Ins2* mRNA and proinsulin were present in the neurons of the hippocampus and cerebral cortex. They also established that insulin secretion from hippocampal neurons was regulated by Ca^2+^-dependent activator protein for secretion 2 (CAPS2). This means that, such as the myriad of other neuropeptides, insulin is secreted from neurons via exocytosis from dense-core vesicles. The treatment of hippocampal neurons with Aβ_1–42_ significantly decreased the amount of proinsulin detected in these cells. Moreover, the Aβ_1–42_ treatment decreased Ser9-phosphorylation of glycogen synthase kinase-3β (GSK-3β). Namely, the binding of insulin to its receptor activates PI3K/Akt cascade, which results in GSK-3β inactivation through Ser9-phosphorylation. When taken together, these data suggest that downregulation of hippocampal *Ins2* expression in Aβ_1–42_-induced model of Alzheimer’s disease occurs via activation of GSK-3β. This conclusion was backed by the fact that the effect of Aβ_1–42_ treatment was reduced by both GSK-3β siRNA and lithium, a mood stabilizer is known to inhibit GSK-3β activity.

In addition to the hippocampus, the significance of insulin expression in the CNS in the context of Alzheimer’s disease was documented in the cerebral cortex as well [19]. This electrophysiological study revealed that adding glucose to the external solution of neocortical brain slices resulted in GABAergic NGFC reversibly decreasing the frequency and amplitude of spontaneous excitatory postsynaptic potentials (EPSP) in neighboring pyramidal neurons via a mechanism that included activation of the insulin receptor. The same effect was achieved by adding exogenous insulin to the brain slices, while the application of insulin receptor antagonists resulted in the prevention of these excitation-suppressing effects on local microcircuits detected after the addition of hyperglycemic solution. Considering that lower cerebral levels of insulin have been associated with neurodegenerative diseases such as Alzheimer’s [77], a potential adaptive significance of insulin expression in NGFC could be a modulation of neighboring neuronal circuits involved in learning and memory.

## 4. Conclusions

Based on the body of evidence gathered thus far, it is safe to say that the actions of insulin produced in the CNS are diverse and vary from one brain region to another. It ought to be underlined that most of the studies cited in this manuscript were performed on rodents whose genome, unlike the human, contains two nonallelic insulin genes. However, despite this limitation, the need for additional characterization of brain insulin expression in regard to human health is important, given that intranasal insulin application has been proven to be successful as a treatment not only for Alzheimer’s disease and mild cognitive impairment but for other complications such as cerebral ischemia, traumatic brain injuries, and postoperative delirium [83]. The efficacy of intranasal insulin treatments suggests that brain-derived insulin may be able to reach some of its effector cells within the CNS, which pancreatic insulin cannot be transported to. Therefore, further identifications of cellular sources and targets of brain-derived insulin and their anatomical position in the brain will help us to better understand its mechanism of action and use that knowledge to prevent and/or treat various neurodegenerative disorders.

## Figures and Tables

**Table 1:** Representative publication confirming the neuronal insulin expression in vitro and in vivo.

In Vitro Studies				
Cell Culture Type	Species		Method	Reference
Primary fetal brain culture	Rat		ICC	Weyhenmeyer and Fellows, 1983 [11]
Neuron-enriched primary cultures	Rat		ICC	Raizada et al., 1983 [12]
Fetal brain cell cultures	Mouse		HPLC and gel filtration	Birch et al., 1984 [13]
Neuronal cell primary culture	Rat		RIA and HPLC	Clarke et al., 1986 [18]
Neuronal cell primary culture	Rabbit		ICC and In Situ Hybridization	Schechter et al., 1988 [16]
Neuronal cell primary culture	Rabbit		ICC, In Situ Hybridization and ELISA	Schechter et al., 1990 [14]
Neuronal cell primary culture	Rabbit		RT-PCR and In Situ Hybridization	Devaskar et al., 1994 [17]
Immortalized embryonic rat hippocampal clonal cell line	Rat		ICC and Southern blot	Singh et al., 1997 [15]
Neuron cell cultures	Rat		RT-PCR and ICC	Schechter et al., 1998 [35]
Neuron cell cultures	Rat		qRT-PCR	Schechter et al., 1999 [36]
mHypoE-39 and mHypoE-46	Mouse		RT-PCR and ICC	Madadi et al., 2008 [21]
Adult neuronal cells derived from hippocampus and olfactory bulbs	Rat		Microarray and ICC	Kuwabara et al., 2011 [37]
mHypoE-39	Mouse		qRT-PCR and IF	Lee et al., 2016 [22]
Primary culture of epithelial cells of the choroid plexus	Mouse		qRT-PCR and IF	Mazucanti et al., 2019 [38]
**In vivo studies**				
**Brain Region**	**Species**	**Age**	**Method**	**Reference**
Whole brain extract; Hypothalamus, olfactory bulb, cerebellum, brainstem, cerebral cortex	Rat	Adult	RIA and IHC	Havrankova et al., 1978 [6]
Neocortex, hippocampus, hypothalamus, thalamus,	Rat	Adult	IF	Dorn et al., 1980 [25]
Cerebellum, brain stem, cortex, hippocampus, thalamus and hypothalamus	Rat and mouse	Adult	IF and RIA	Dorn et al., 1981 [24]
Hypothalamus, hippocampus, and olfactory bulbs	Rat (female)	Adult	RIA	Baskin et al., 1983 [26]
Hypothalamus	Human	Adult	IF	Dorn et al., 1983 [27]
Whole brain	Human, rat, mouse, tortoises, and frogs	Adult	RIA and IHC	Dorn et al., 1983 [39]
Hippocampus, hypothalamus and brainstem	Human	20–25-week embryos and adult	IHC	Dorn et al., 1984 [28]
Hypothalamus, olfactory bulbs, hippocampus, amygdale, cortex, midbrain, and hindbrain	Rat	2–3 months old	RIA	Baskin et al., 1985 [7]
Hypothalamu (PEV)	Rat	Adult	In Situ Hybridization	Young, 1989 [40]
Whole brain	Rabbit	Fetal, neonatal and adult	ELISA, Western blot, RIA, Northern blot and HPLC	Schechter et al., 1992 [29]
Whole brain	Rat	Fetal, neonatal and adult	RT-PCR	Devaskar et al., 1993 [30]
Whole brain	Rat	Embryos	RT-PCR	Deltour et al., 1993 [41]
Hippocampus and olfactory bulbs	Rabbit	/	RT-PCR and In Situ Hybridization	Devaskar et al., 1994 [17]
Whole brain	Rat	Embryos	PCR and electron microscopy	Schechter et al., 1996 [31]
Cortex	Human (male and female)	Middle-aged, aged and Alzheimer’s	RIA and IHC	Frölich et al., 1998 [42]
Whole brain	Drosophila	/	qRT-PCR	Hwangbo et al., 2004 [43]
Hypothalamus, hippocampus, cortex	Human	Adult and Alzheimer’s	qRT-PCR	Steen et al., 2005 [44]
Cerebellum	Rat	Neonatal	qRT-PCR	de la Monte et al., 2005 [45]
Front parietal cortex, hippocampus and hypothalamus	Rat	3–4 months	qRT-PCR	Grünblatt et al., 2007 [46]
Hypothalamus, telencephalon, thalamus, brainstem, visual cortex, and cerebellum	Nile Tilapia	Adult	qRT-PCR, In situ hybridization and IHC	Hrytsenko et al., 2007 [47]
Cortex	Rat	12 months	qRT-PCR	Osmanovic et al., 2010 [48]
Hippocampus	Rat (female)	7–12 weeks	In situ hybridization and IHC	Kuwabara et al., 2011 [37]
Cerebellum, cortex, olfactory bulb, hippocampus	Mouse	3 and 6 months	qRT-PCR	Mehran et al., 2012 [32]
Cortex (GABAergic interneurons)	Rat	3–5 weeks	single-cell digital PCR	Molnár et al., 2012 [19]
Hippocampus and cortex	Rat	/	RT-PCR, Western blot and IHC	Nemoto et al., 2014 [49]
Hypothalamus	Mouse	8 weeks	qRT-PCR and IF	Lee et al., 2016 [22]
Hypothalamus (PEV)	Rat	2 months	qRT-PCR, RIA, IHC, IF and Western blot	Dakic et al., 2017 [50]
Cortex	Rat	3–5 weeks	single-cell digital PCR	Csajbók et al., 2019 [20]
Choroid plexus	Mouse	/	FISH, IF, qPCR and IHC	Mazucanti et al., 2019 [38]
Hypothalamus (PVN)	Mouse	8–12 weeks	In situ hybridization and IF	Lee et al., 2020 [51]
Hypothalamus (PEV)	Rat (female)	2 months	qRT-PCR, RIA and IHC	Dakic et al., 2022 [52]

Abbreviations: RIA—radioimmunoassay; ICC—immunocytochemistry; IF—immunofluorescence; IHC—immunohistochemistry; FISH—fluorescence in situ hybridization; PEV—periventricular nucleus; PVN—paraventricular nucleus.

## Data Availability

Not applicable.
